# Periodical assessment of four horns of knee meniscus using MR T2 mapping imaging in volunteers before and after amateur marathons

**DOI:** 10.1038/s41598-022-16000-0

**Published:** 2022-07-15

**Authors:** Xuesong Zhang, Jujia Li, Congcong Ren, Ping Zhang, Yan Zeng, Ranxu Zhang, Ming Wang, Xiaoyue Zhou, Jian Zhao

**Affiliations:** 1 Department of Radiology, The Third Hospital of Hebei Medical University, Hebei Province Biomechanical Key Laboratory of Orthopedics, Shijiazhuang, 050051 Hebei China; 2 Siemens Healthineers, Ltd., Shanghai, 201318 China

**Keywords:** Diseases, Medical research

## Abstract

To observe the changes and recovery of T2 values of menisci in amateur marathon participants at different times, and to examine the effect of marathon exercise on meniscal microstructure. Twelve healthy marathon volunteers were recruited continuously, including 5 males and 7 females, with mean (± SD) age of 27.5 ± 5.2 years. The body mass indices (BMIs) ranged from 17.6 to 27.2 kg/m^2^, with a mean of 21.9 ± 2.5 kg/m^2^. The 24 knee joints were scanned using a 3 T MR scanner at 1 week before the event, and at 12 h and 2 months after the event. T2 values of the anterior horn of the medial meniscus (MMAH), posterior horn of the medial meniscus (MMPH), anterior horn of the lateral meniscus (LMAH), and posterior horn of the lateral meniscus (LMPH) were measured by drawing the regions of interest (ROIs) on the T2 map images. Wilcoxon sign rank test was used to compare the T2 values between 1 week before and 12 h after the event, and between 1 week before and 2 months after the event in each anatomical region, respectively. The T2 values of the menisci at 12 h after the event were significantly higher (*P* < 0.05) than those at 1 week before the event. No statistically significant differences in the T2 values of the menisci were found between 2 months after and 1 week before the event (*P* > 0.05). The T2 values of MMAH, MMPH, LMAH, and LMPH showed a trend of "increasing first and then decreasing" over time, suggesting that the T2 values may reflect meniscal microstructure in amateur marathon runner.

## Introduction

Marathon running is a popular recreational activity involving an estimated 30 million people in the United States. Given this sport’s repetitive loading of the knee joint^1–3^, marathon running may affect the condition of the cartilage, meniscus, and other relevant tissues. The meniscus is an integral part of the knee and is considered the second stabilizer of the knee joint^4,5^, playing an important role in shock absorption, joint stability, joint lubrication, load transmission, and maintenance of articular cartilage integrity^6–8^. The meniscus is also one of the most frequently injured parts of the knee joint^5,9,10^, so the integrity of the meniscus—in both shape and function—is critical for the long-term health of the knee^6,8,11^.

There is ongoing interest in assessing whether marathon running could ultimately lead to unrecoverable knee injury. However, the effect of running on degenerative disease is still controversial^2^. Knee osteoarthritis (OA) is a disease characterized by changes in articular cartilage and subchondral bone structure. The prevalence of knee OA is increasing significantly, arousing people more attention to the health of knee^12^. Meniscal lesions such as meniscal degeneration and meniscal tears have been identified as strong determinants within the multifactorial etiology of knee osteoarthritis^7,11,13^. Hence, it would be clinically significant if there is a correlation between marathon running exercise and the physiologic state and injury of the knee meniscus. Recent studies have shown that quantitative magnetic resonance imaging (MRI) methods, such as T1/T2/T2* mapping or T1rho mapping, are helpful for continuous assessments of meniscal conditions and early detection of meniscal degeneration^3,5–8,14–16^. Furthermore, MRI has become integral for the diagnosis and treatment strategy of knee pathology^17^. There was study that has shown T1*ρ* may be more suitable to measure the dynamic changes in articular cartilage, while T2 may be more appropriate for measuring the dynamic changes in the meniscus associated with OA progression^6^. Among various quantitative methods, T2 mapping has been shown to provide enhanced morphologic and biochemical assessments of soft tissue structures, including the menisci^11^. In the present study, the T2 values of menisci were obtained at three distinct time points in volunteers who are marathon runners; these values were then analyzed to detect meniscal changes and assess the effects of marathon running on the meniscal microstructure.

## Materials and methods

### General information

Fifteen volunteers who were amateur marathon runners were recruited continuously. They all finished the half marathon. According to the inclusion and exclusion criteria, 3 volunteers did not meet the requirements; therefore, MRI examinations were performed on 12 eligible volunteers in this study, across a total of 24 knee joints. Two volunteers were lost to follow-up 2 months after the marathon, so the number of knee joints 2 months after the event was 20. The 12 eligible volunteers consisted of 5 males and 7 females. Their ages ranged from 21 to 37 years, with a mean ± standard deviations of 27.5 ± 5.2 years. Body mass indices (BMIs) ranged from 17.6 to 27.2 kg/m^2^, with a mean of 21.9 ± 2.5 kg/m^2^. The mean running frequency of all volunteers exceeded 3 times per week, with a mean distance and duration of 5 km and 30 min each time. The 24 knee joints of the volunteers were subjected to MRI examination 1 week before, and 12 h and 2 months after the marathon. The timing of 2 months after the marathon was defined within 60–65 days. We confirm that all methods were performed in accordance with the relevant guidelines and regulations.

### Inclusion and exclusion criteria

Inclusion criteria: (1) Age 20–40 years; (2) BMI < 30 kg/m^2^; (3) Had not participated in a marathon in the preceding 6 months; (4) Running less than 20 km per week; (5) No history of knee injury, surgery, or infection; (6) No positive signs of knee joint injury in the preceding 5 years; (7) No history of chronic diseases requiring medical treatment; and (8) No contraindication for an MRI examination.

Exclusion criteria: (1) Serious artifacts observed in MR images, precluding the accurate T2 value measurements; (2) Routine MRI images of the knee joint before running showed meniscal injury; or (3) Volunteers not available at 12 h after the marathon.

### MR scanning method and sequence parameters

All the volunteers underwent MRI on a 3.0-T scanner (MAGNETOM Verio, Siemens Healthcare, Erlangen, Germany) with an 8-channel phased-array knee coil. Scanning protocols were as follow: (1) Sagittal fat-suppressed proton density weighted imaging (FS‐PDWI): TR, 3500.0 ms; TE, 24.0 ms; FOV, 180.0 mm × 180.0 mm; flip angle, 150°; and slice thickness, 4.0 mm; (2) The multiple-TE fast spin echo sequence was used to evaluate T2 maps as follows: TR, 3410.0 ms; TE, 13.8, 27.6, 41.4, 55.2, and 69.0 ms; FOV 160.0 mm × 160.0 mm; flip angle, 180°; and slice thickness, 2.7 mm. All volunteers rested for 1 h before each scan and were examined in a supine position. Participants were asked to keep their lower limbs naturally straight, the lower edge of the patella was used as the scanning center, and the knee joint under examination was fixed with sandbags and sponges in the space between the patella and the coil as well as at the ankle to minimize inconsistent joint angles and motion artifacts. In order to ensure the consistency of scanning position, the scanning parameters, including the thickness and slices of image reconstruction, were set to the same.

## Image analysis

**Figure 1:**
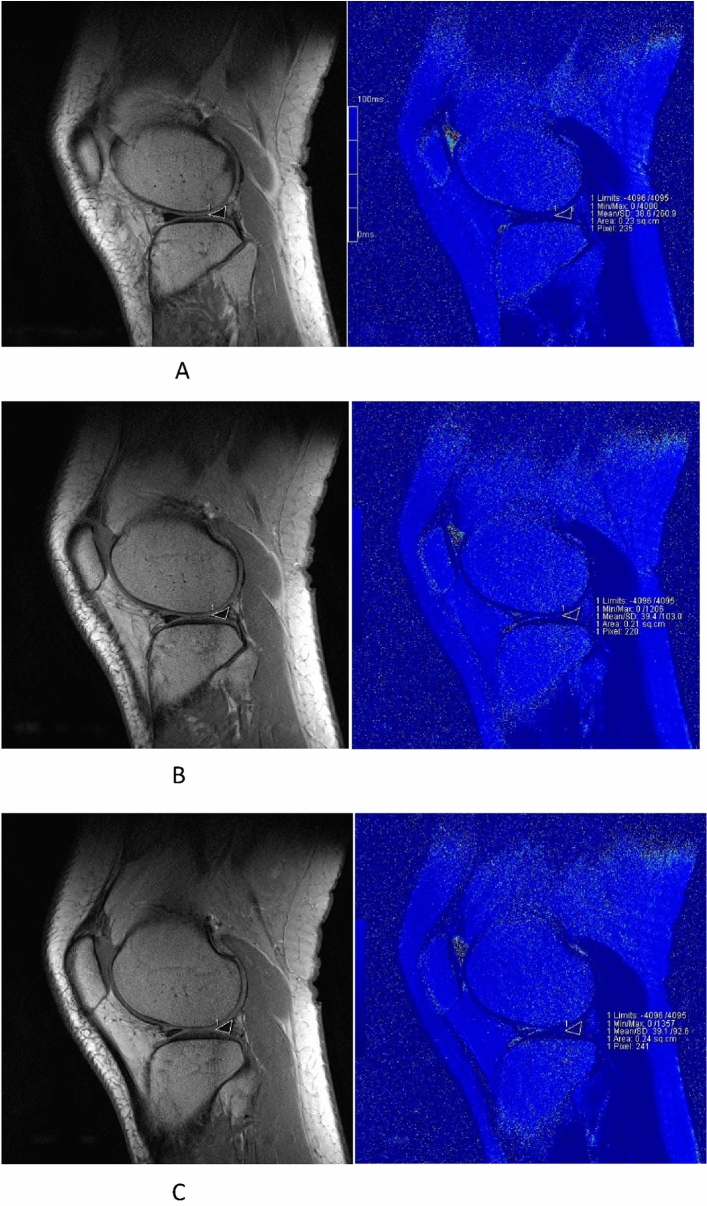
Representative images for image analysis. These figures show the left knee in a 23-year-old male before the marathon. pseudo-color images were synchronized with the anatomical images. Three adjacent maximum planes of LMPH were selected; the regions of interest were delineated manually along the edge of the meniscus on the anatomical images; and T2 values were obtained via T2 mapping, accordingly. (**B**) is the medial plane; (**A**) and (**C**) are adjacent planes of (**B**). The T2 values were 38.6 ms, 39.4 ms, 39.1 ms, respectively.

All raw data was transferred to a Siemens workstation (Software NUMARIS/4, Version syngo MR B19) after the images were collected. The meniscus was divided into four subregions, corresponding to MMAH, MMPH, LMAH, and LMPH. The images were analyzed independently by two diagnostic radiologists who each had 5 or more years of experience in diagnostic musculoskeletal imaging. The images were analyzed as follow: Synchronize the anatomical images with the pseudo-color images, three adjacent planes with the largest area of the meniscus in each subregion were displayed, and then the ROI along the edge of the meniscus was delineated manually on the anatomical images, the same ROI contour will appear on the corresponding pseudo-color images. T2 values were obtained in each plane in the pseudo-color images; representative images are presented in Fig. 1. The mean value of the three measurements was taken as the T2 value of the corresponding subregion. To reduce measurement errors, surrounding tissues (e.g., cartilage, ligaments, and joint effusion) were avoided when outlining the ROI.

## Statistical analysis

Statistical analyses were conducted using SPSS 21.0 statistical software. The consistency of the data measured by the two radiologists was tested for reproducibility using the intraclass correlation coefficient (ICC). For each subregion, wilcoxon sign rank tests were used to compare the T2 values of menisci from 1 week before the event to those collected at 12 h and 2 weeks after the event because of the absent normal distribution. Analysis used an α of 0.05; *P* values < 0.05 were considered statistically significant.

### Ethical approval

This study was approved by the Ethics Committee of the Third Hospital of Hebei Medical University, and all participants gave informed consent before taking part.

### Informed consent

Informed consent was obtained from all individual participants included in the study.

## Results

**Figure 2:**
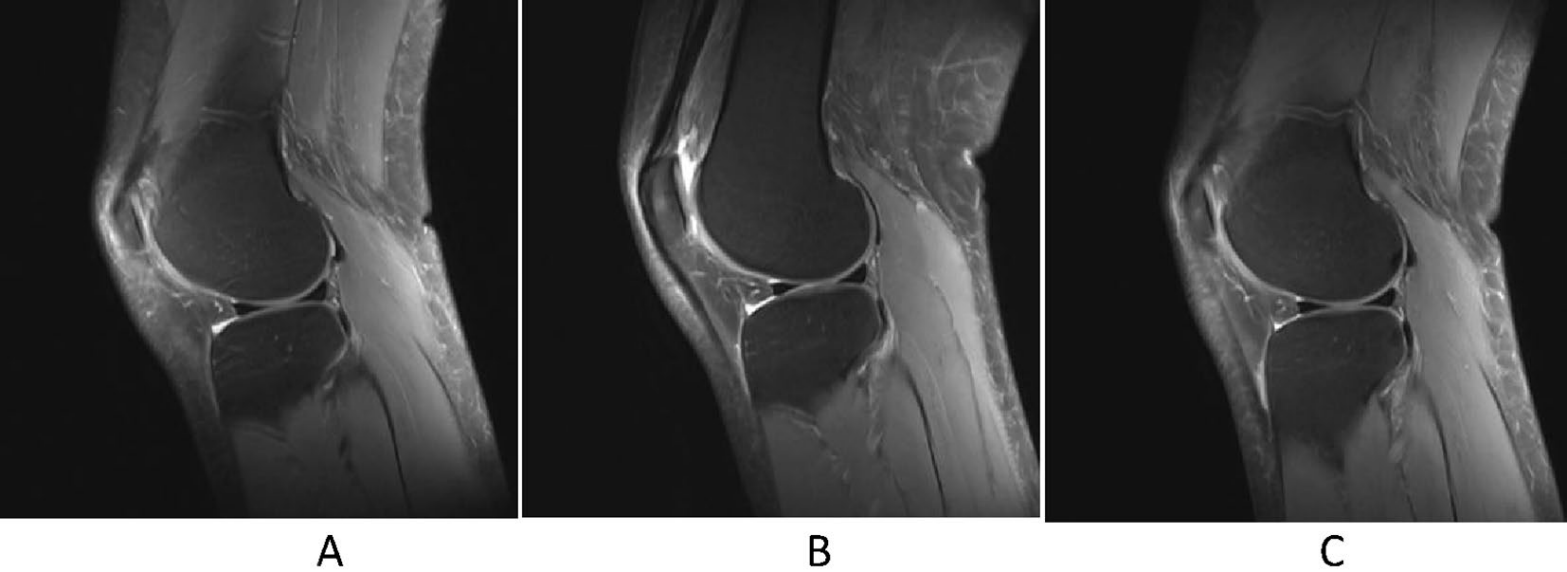
Representative images demonstrating the state of the meniscus and cartilage. Specifically, these FS‐PDWI images of the left knee in a 28-year-old female show that the cartilage was continuous and complete with a smooth surface, lacking obvious abnormal signal changes, meniscal tears, ligament or tendon injuries, subchondral bone changes, free bodies, or joint effusions when assessed 1 week before the marathon (**A**) and 12 h (**B**) or 2 weeks (**C**) after marathon running.

Anatomic imaging by FS‐PDWI showed that the knee cartilage of all the volunteers was continuous and complete, consisting of a smooth surface both before and after running. The alignments of knee were less than 5° for all the volunteers. There were no obvious abnormal signal changes in the cartilage of the volunteers; no meniscal tears, ligament or tendon injuries, subchondral bone changes, free bodies, or joint effusions were apparent (Fig. 2).

### Data reproducibility tests

Consistency tests were performed for the mean values determined in each region by the two radiologists and test–retest (Table 1). The relative strength of consistency was defined as poor (ICC < 0.40), average (ICC: 0.40 ≤ ICC < 0.60), good (ICC: 0.60 ≤ ICC < 0.75), or excellent (ICC ≥ 0.75). In this study, the measured consistency range was 0.764 to 0.956, indicating good consistency and reproducibility.

**Table 1:** The ICC of T2 values in bilateral menisci as independently measured by two radiologists.

ICC	1 week before event	Within 12 h after event	2 months after event
Two radiologists	0.956	0.866	0.878
Test–retest	0.916	0.764	0.782

*ICC* intraclass correlation coefficient.

### Changes in T2 values in different subregions over time

The T2 values of MMAH, MMPH, LMAH, and LMPH at 1 week before the run were compared with the values for the respective regions at 12 h and 2 months after the event. The results showed that the T2 values of menisci at 12 h after the event were significantly (*P* < 0.05) higher than those before the run. In contrast, no statistically significant differences in the meniscal T2 values were detected when compared between images obtained 2 months after the event and 1 week before the run (*P* > 0.05).

1. A comparison of T2 values in each subregion between 1 week before and 12 h after a run.

Wilcoxon sign rank tests were performed on the T2 values of menisci from 12 volunteers because of the absent normal distribution, permitting a comparison of images collected at 1 week before and 12 h after the run. As shown in Table 2, the T2 values of MMAH, MMPH, LMAH, and LMPH 12 h after the event were significantly (*P* < 0.05) higher than the respective values before a run.

(2) A comparison of T2 values in each subregion between 1 week before and 2 months after a run

**Table 2:** Comparison of T2 values in each subregion at different timings [M (P25,P75)] (ms).

	MMAH	MMPH	LMAH	LMPH
Timing 1	34.6(32.2,38.7)	34.3(30.7,42.1)	33.1(29.5,41.0)	32.6(30.0,41.9)
Timing 2	46.6(35.9,58.0)	39.1(35.0,55.5)	41.6(28.7,57,1)	44.2(34.6,53.4)
Timing 3	40.8(37.6,48.5)	34.9(32.1,38.7)	37.6(30.7,41.4)	36.0(32.8,41.7)
P (1 vs. 2)	0.003*	0.008*	0.024*	0.002*
P (1 vs. 3)	0.055	0.513	0.232	0.391

*Statistically significant difference (*P* < 0.05).

Two volunteers were lost at follow-up 2 months after the marathon run; therefore, only 20 knee joints were included in the comparison data. Wilcoxon sign rank tests were performed on the T2 values of menisci because of the absent normal distribution between 1 week before and 2 months after the run. As shown in Table 2, differences between the T2 values of the respective regions (MMAH, MMPH, LMAH, and LMPH) were not significantly different when 2 months after (timing 3) and 1 week before the marathon run (timing 1) were compared (*P* > 0.05).

## Discussion

In this study, we made a periodical assessment of MMAH, MMPH, LMAH, and LMPH of knee meniscus using T2 mapping in volunteers at three timings. We observed that the T2 values of the four horns 12 h after the event were significantly (*P* < 0.05) higher than the respective values before a run, and differences between the T2 values of the respective regions were not significantly different when 2 months after and 1 week before the marathon run were compared (*P* > 0.05), leading T2 values in menisci to show a general trend of "first increasing and then decreasing".

Meniscal function is heavily dependent on the properties of the extracellular matrix (ECM). In a normal meniscus, the ECM is composed of 72% water, 22% collagen, and 0.8% glycosaminoglycan, but these components can vary with age, sex, etc.^7,8,10,15^. The bunching of collagen fibers, primarily Type I, within the meniscus determines the tensile strength and shock-absorbing properties of the tissue, while proteoglycans contribute to compressive strength by upholding swelling pressure^8^. For evaluation of menisci, MRI is considered to be the best non-invasive imaging method, given that this technique is able to assess differences in the biochemistry of menisci^5^. As an indirect biomarker of meniscal structure, T2 value provides information about the interaction between water molecules and the ECM, especially given that T2 analysis is based on the interactions among collagen content, direction, and anisotropy, permitting more accurate analysis of potential changes in menisci^5,8,15^. T2 mapping MRI are capable of detecting meniscus degeneration and injury quantitatively^18^, which can be implemented in a clinical environment without requiring hardware modifications^19^.

**Figure 3:**
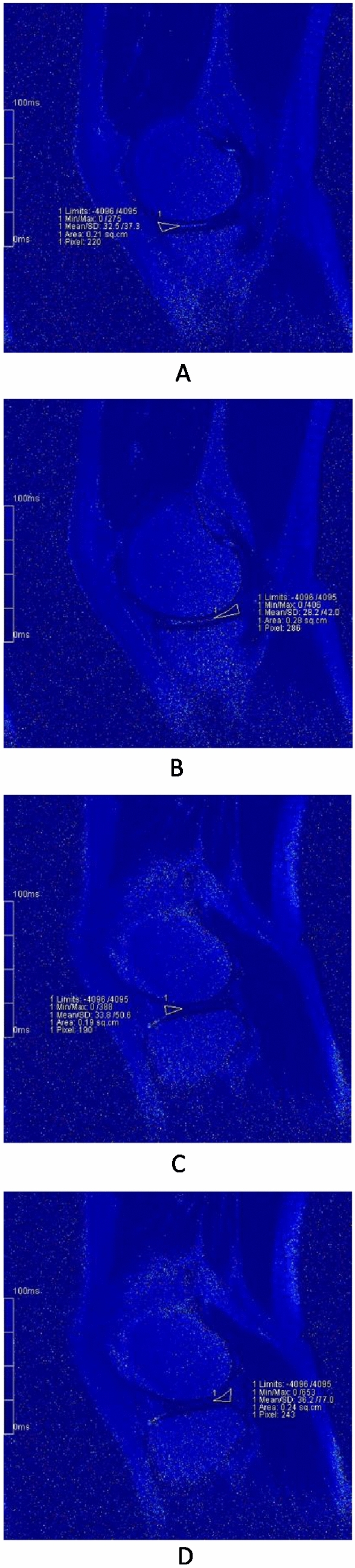
The pseudo-color images of left knee of a 28-year-old female at three timings. (**A**)–(**D**) are the images before the match. Fig. A/B/C/D are the images MMAH/MMPH/LMAH/LMPH respectively. The T2 values accordingly were 32.5 ms, 28.2 ms, 33.8 ms, 36.2 ms, respectively.

**Figure 4:**
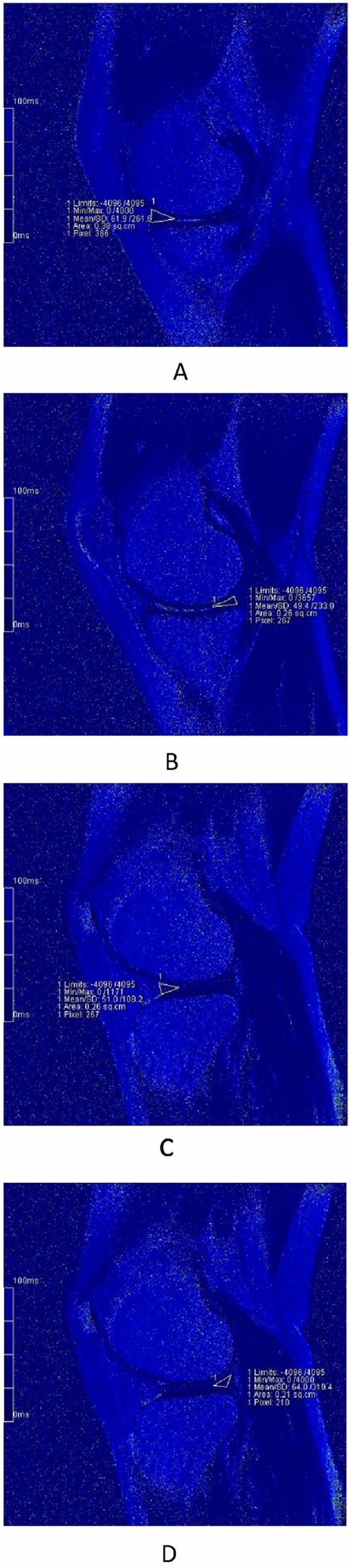
The pseudo-color images of left knee of a 28-year-old female at three timings. (**A**)–(**D**) are the images 12 h after the match. Fig. A/B/C/D are the images MMAH/MMPH/LMAH/LMPH respectively. The T2 values accordingly were 61.9 ms, 49.4 ms, 51.0 ms, 64.0 ms, respectively.

**Figure 5:**
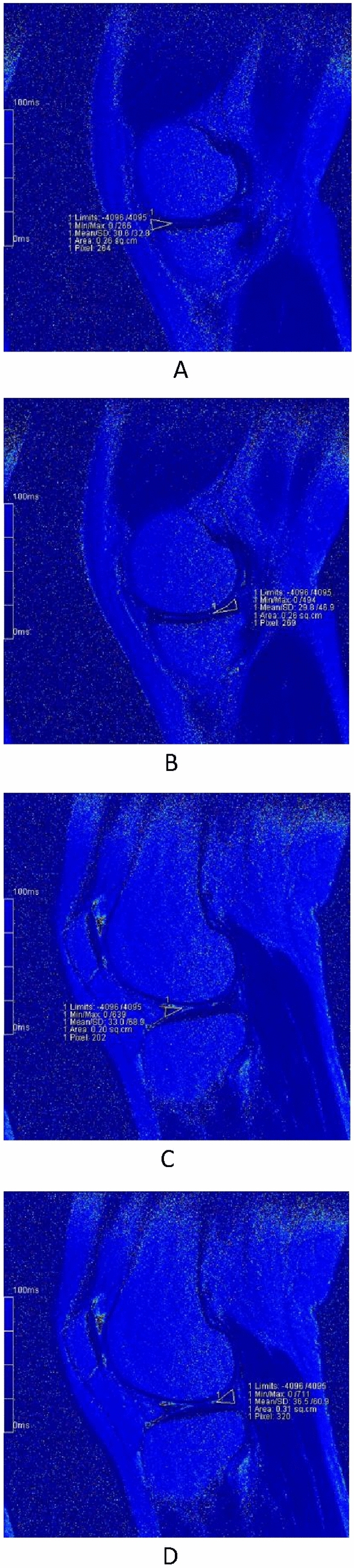
The pseudo-color images of left knee of a 28-year-old female at three timings. (**A**)–(**D**) are the images 2 months after the match. Fig. A/B/C/D are the images MMAH/MMPH/LMAH/LMPH respectively. The T2 values accordingly were 30.6 ms, 29.8 ms, 33.0 ms, 36.5 ms, respectively.

Our results revealed that T2 values of menisci were significantly higher at 12 h after a marathon compared to values at 1 week before the run (Figs. 3A-D and 4A-D), suggesting an acute change in the collagen and water content, parameters that are detected by T2 mapping. Interestingly, the condition of menisci had recovered by 2 months after the event, returning to T2 values that did not differ significantly from those obtained at baseline (1 week before the run) (Figs. 3A-D, 5A-D). This observation indicated that T2 values may be of use in evaluating menisci, a result that is consistent with those of previous studies^3,6,13,16,19^. Some studies achieved good results by also using T1ρ to evaluate the menisci^6–8,20^. However, T2 may be more appropriate for measuring dynamic changes in the menisci associated with OA progression, whileT1ρ may be more suitable for measuring dynamic changes in articular cartilage^6^. On the other hand, our results also suggested that marathon exercise does not cause unrecoverable damage to the meniscus. Indeed, several other studies support this inference, suggesting that the running of marathons may not have a harmful effect on knee joints and may not predispose participants to the development of premature OA^1,2,21,22^. Separate from effects on the meniscus, the running of marathons may not have adverse effects on articular cartilage, as suggested by our previous study^23^.

Stehling et al.^5,7^ studied the T2 values of menisci after a marathon, showing that the T2 values of all regions of menisci increased significantly (*P* < 0.0001) in all surveyed marathon volunteers (n = 9) following a race, suggesting that the biochemical composition of the menisci may have changed in the knees of these runners. In the present study, we found that the T2 values of menisci also were elevated (compared to a pre-dose baseline) at 12 h after the event, although the P values in our study were not as robust as those in the study of Stehling et al., possibly due to the small sample size.

There is an internal relationship between the structure and function of knee cartilage and the meniscus. Multiple factors influence this interaction, and it is difficult to determine the causal nature of this relationship. Nonetheless, patients with meniscal tears have been shown to exhibit an increased risk of cartilage injury^4,6^. Using MRI to evaluate the effects of long-distance running on lower extremity joints, Qiu et al.^16^ found that previously uninjured joints and short-term follow-up did not show obvious intrachondral disorders, consistent with the lack of apparent effects on menisci at 2 months in the present study. Unfortunately, the post-run MRI results in the study of Qiu et al. were obtained at a range of intervals (2.5 to 14.0 days post-event), rather than at a single specified time, and the post-run recovery data were only partially recorded. These facts make it difficult to ascertain, from that previous study^5^, how much time was needed for complete recovery, and indeed whether recovery actually occurred. Thus, it remains unclear whether marathon-like exercise exerts consistent effects on cartilage and menisci, and whether these tissues can recover over time.

When measuring the menisci, we found the T2 values were difference between the left and right knees for some volunteers, although there is no overall difference. Therefore, we included bilateral menisci in the observation, which may be closer to the actual results. We speculate that the cause of the discrepancy is even on the same volunteer, the morphology and position of bilateral menisci are not exactly the same^24^, the loading and impact on which will not be exactly the same, likewise.

In the present study, although T2 values in menisci showed a trend of "first increasing and then decreasing", this change was not observed consistently in every subject. In some volunteers, the T2 values at the 3 time points showed progressive increases, progressive decreases, initial decreases followed by increases, initial increases followed by decreases, lack of apparent changes, etc. These various phenomena may reflect individual differences among the volunteers. Adaptive changes may have taken place in the knee joints of some subjects who were in better physical condition and persistent exercisers, thereby ensuring that long-distance running did not cause acute meniscal change. That is, for some subjects, dynamic changes would not have been apparent in the knee joint, and the T2 values at 12 h after the event even may be slightly lower than those at baseline. For some subjects, the emergent state after the run may be obvious and lasting, and the recovery state may be achieved very slowly, such that the "inflection point" of recovery had not been reached in our second follow-up. Under such circumstances, T2 values in menisci at 12 h post-run would be obviously increased compared those measured at 1 week before the event, while T2 values in menisci 2 months post-run would remain similar to those observed at 12 h after the marathon.

In addition, the increase in T2 values in menisci 2 months post-marathon (compared with values at 1 week pre-run) was observed primarily in LMPH. We speculate that the follow-up time was not sufficient to definitively detect meniscal injuries or degeneration in this region. There are two reasons for this hypothesis. First, we did not find clear abnormal imaging evidence of meniscal tears in the original images. Second, the literature suggests that, even if the structure and function of meniscal changes, these changes occur primarily in the MMPH^6,8,14,25^, while the LMPH is rarely involved. The medial meniscus is less active, larger in diameter, and thinner than other meniscal regions, and therefore, might be more susceptible to injury^6^. Even if LMPH injuries do occur, such lesions do not appear to have clinical significance, given the rarity of surgeries for repair of lateral meniscal injuries^4^.

Previous studies proposed T2 values to evaluate the cartilage^16,26^. There has been study suggesting an increase in T2 relaxation time of cartilage, other study, however, suggested the condition of the articular cartilage might achieved sustained improvement for at least 6 months post-marathon^16,22^. But there is rarely study focused on T2 value change of meniscus independently. At present, many studies related to the meniscus divide the meniscus into three parts of the same width, based on the characteristics of the blood supply to the meniscus^25^. However, to our knowledge, there have not been studies examining dynamic changes in the meniscus over time that have divided the meniscus into quadrants (i.e., medial and lateral regions overlapping with anterior and posterior regions). For most studies, follow-up from long-distance running events such as marathons typically is limited to evaluation at a single time point, and such follow-ups often are performed very soon after the event. These limitations are expected to preclude the detection of dynamic changes in the meniscus, and indeed, research evaluating the recovery of the meniscus after longer intervals appear to be absent from the literature.

The present study had several limitations. First of all, the sample size was small, limiting the robustness of our results. And we included bilateral menisci in the observation, which would overestimate the statistical analysis to some extent. Secondly, we remain unsure as to whether it is reasonable to set the second follow-up time at 2 months post-run. Given the lack of evidence that the meniscal condition does not change again, it might be better to obtain the T2 values 6 months post marathon running^22^. Thirdly, we studied T2 values of the horns without the body of the menisci. In addition, we didn’t take clinical demographics (such as knee alignments and body weight), the cartilage covered and uncovered by meniscus^27^, or duration of marathon experience into consideration. In future research, we intend to increase the sample size and prolong the follow-up time.

## Conclusions

In conclusion, T2 mapping plays an important role in assessing meniscal microstructure changes. T2 values for the menisci of amateur marathon runner volunteers generally showed a trend of "increasing first and then decreasing", as assessed at three time points (1 week before, and again 12 h and 2 months after marathon running). Therefore, we speculate that the menisci might recover to the pre-run levels in the long-term (i.e., months). These results also suggest that clinicians and radiologists should consider time since a running event as a specific factor in evaluating patients; this consideration could facilitate a decrease in the number of misdiagnoses.
